# Supplementary material for: Full spectrum fluorescence lifetime imaging with 0.5 nm spectral and 50 ps temporal resolution
Source: Nat Commun. 2021 Nov 16;12:6616. doi: 10.1038/s41467-021-26837-0 (PMC8595732; doi:10.1038/s41467-021-26837-0)
Supplement: Supplementary file 1 — Reporting Summary [file 41467_2021_26837_MOESM1_ESM.pdf]

nature portfolio

Corresponding author(s): Mark Bradely, John Girkin

Last updated by author(s): Oct 27, 2021

Reporting Summary

Nature Portfolio wishes to improve the reproducibility of the work that we publish. This form provides structure for consistency and transparency in reporting. For further information on Nature Portfolio policies, see our Editorial Policies and the Editorial Policy Checklist.

Statistics

For all statistical analyses, confirm that the following items are present in the figure legend, table legend, main text, or Methods section.

n/a	Confirmed	
☐	☒	The exact sample size (n) for each experimental group/condition, given as a discrete number and unit of measurement
☐	☒	A statement on whether measurements were taken from distinct samples or whether the same sample was measured repeatedly
☒	☐	The statistical test(s) used AND whether they are one- or two-sided<br>*Only common tests should be described solely by name; describe more complex techniques in the Methods section.*
☒	☐	A description of all covariates tested
☐	☒	A description of any assumptions or corrections, such as tests of normality and adjustment for multiple comparisons
☐	☒	A full description of the statistical parameters including central tendency (e.g. means) or other basic estimates (e.g. regression coefficient) AND variation (e.g. standard deviation) or associated estimates of uncertainty (e.g. confidence intervals)
☒	☐	For null hypothesis testing, the test statistic (e.g. F, t, r) with confidence intervals, effect sizes, degrees of freedom and P value noted<br>*Give P values as exact values whenever suitable.*
☒	☐	For Bayesian analysis, information on the choice of priors and Markov chain Monte Carlo settings
☒	☐	For hierarchical and complex designs, identification of the appropriate level for tests and full reporting of outcomes
☒	☐	Estimates of effect sizes (e.g. Cohen's d, Pearson's r), indicating how they were calculated

Our web collection on statistics for biologists contains articles on many of the points above.

Software and code

Policy information about availability of computer code

Data collection	Data was collected using Matlab (2019b) scripts that communicated with the detector FPGA via firmware interfacing through the Opal Kelly frontpanel driver (version 4.5.6.0). The firmware bit-file enabled control of the on-PCB digital to analog converters which provides drive signals to the galvanometric mirrors. Data was streamed from the image sensor to the FPGA to the PC running Matlab code. Retrieved data was was entered into a four dimensional array (X, Y, Time bin, Spectral channel) which is saved as a Matlab file for image processing along with appropriate system perimeters such as selected image size and the length of the time bins determined by the "histmode" parameter with code provided through the University of Edinburgh Datashare facility, https://doi.org/10.7488/ds/3099
Data analysis	Data was analyzed using Matlab (2019b) scripts that take the 4D array created by the collection script as an input along with saved metadata such as frame size and time bin duration. The analysis scripts allow for user selection of the spectral pixel range to be analyses, the time bins to be taken into account for lifetime fitting, any averaging that should be applied and various plotting perimeters for visualizing data such as the number of wavelengths to plot. Lifetime fitting is performed via least squares using the opensouce algorithm CPUfit (https://github.com/gpufit/Gpufit, Version 1.1.0). The analysis scripts output, along with figures of the selected wavelengths, datacubes containing lifetime and intensity data for the wavelength spectrum. Region of interest analysis was performed via additional scripts that use these lifetime and intensity datacubes as an input and allows the user to import 2 datacubes along with two standard images, select 3 regions of interest (ROI) and output the regions intensity and lifetime spectra along with overlays of the RIOs on the imported images, intensity and lifetime images. Code for analysis is provided with example data, code provided through the University of Edinburgh Datashare facility, https://doi.org/10.7488/ds/3099

For manuscripts utilizing custom algorithms or software that are central to the research but not yet described in published literature, software must be made available to editors and reviewers. We strongly encourage code deposition in a community repository (e.g. GitHub). See the Nature Portfolio guidelines for submitting code & software for further information.

Data

Policy information about availability of data

All manuscripts must include a data availability statement. This statement should provide the following information, where applicable:

- Accession codes, unique identifiers, or web links for publicly available datasets
- A description of any restrictions on data availability
- For clinical datasets or third party data, please ensure that the statement adheres to our policy

Data underling Figures 2-5 and custom code used to analyze the FS-FLIM cubes is available through the University of Edinburgh DataShare facility. https://doi.org/10.7488/ds/3099

Field-specific reporting

Please select the one below that is the best fit for your research. If you are not sure, read the appropriate sections before making your selection.

- [x] Life sciences
- [ ] Behavioural & social sciences
- [ ] Ecological, evolutionary & environmental sciences

For a reference copy of the document with all sections, see nature.com/documents/nr-reporting-summary-flat.pdf

Life sciences study design

All studies must disclose on these points even when the disclosure is negative.

Sample size	The purpose of the work is to highlight the potential types of data output from the system, the sample size for each shown example is 1.
Data exclusions	No data was excluded
Replication	The system output was verified using standard samples well reported in the literature (for example fluorescien solutions) prior to the commencement of measurement of the samples shown. Each sample was measured at least 3 times to verify consistency of result, with the exception of the fresh tissue sample that had to be returned for disposal. All replicate measurements were successful at reproducing the spectral lifetime datasets.
Randomization	As mentioned previously the sample size for each type is 1 and therefore no randomization was used. The aim of the work is to portray the type of information that the optical system can produce, not provide an understanding of the underlying biological mechanisms behind the optical signatures that are shown, for this purpose it it not necessary to have large sample sizes, only to show datacubes are consistent.
Blinding	As the aim of the work is to portray the type of information that the optical system can produce, not provide an understanding of the underlying biological mechanisms behind the optical signatures. Therefore only one sample of each type was required, to produce a representative dataset of the the underlying sample type.

Reporting for specific materials, systems and methods

We require information from authors about some types of materials, experimental systems and methods used in many studies. Here, indicate whether each material, system or method listed is relevant to your study. If you are not sure if a list item applies to your research, read the appropriate section before selecting a response.

Materials & experimental systems

n/a	Involved in the study	
[x]	[ ]	Antibodies
[x]	[ ]	Eukaryotic cell lines
[x]	[ ]	Palaeontology and archaeology
[x]	[ ]	Animals and other organisms
[ ]	[x]	Human research participants
[x]	[ ]	Clinical data
[x]	[ ]	Dual use research of concern

Methods

n/a	Involved in the study	
[x]	[ ]	ChIP-seq
[x]	[ ]	Flow cytometry
[x]	[ ]	MRI-based neuroimaging

Human research participants

Policy information about studies involving human research participants

Population characteristics	The patient that was consented for treatment was a naive 67 year old female with pT3N0M0 poorly differentiated adenocarcinoma who was undergoing curative intent surgery. Subsequent molecular testing revealed no EGFR mutation, ALK or ROS1 gene rearrangement and the tumour cells had no membranous PD-L1 expression. Subject gave written consent.
Recruitment	Participants were recruited to the study if they had a confirmed or suspected lung cancer of >3cm for which curative surgery was being planned. Therefore this specimen represents early stage lung cancer. Macroscopic areas of tumour and normal tussue were dissected by a pathologist and provided for research. The specimen provided internal control of normal and transitional tissue within the assessed sample. Following imaging the sample was fixed and confirmation of areas by immunohistological analyses. For this report we demonstrate a single patient sample. As the aim of the work is to portray the type of information that the optical system can produce, not provide an understanding of the underlying biological mechanisms behind the optical signatures, or provide an actionable assessment of tissue type, bias in patient selection would not impact the result.
Ethics oversight	A favourable ethical opinion was received from the South East of Scotland Research Ethics Service REC 1 (on behalf of the National Health Service), held by the NHS Lothian NRS BioResource (REC ref: 13/ES/0126 and 15/ES/0094).

Note that full information on the approval of the study protocol must also be provided in the manuscript.
