# Supplementary material for: Full spectrum fluorescence lifetime imaging with 0.5 nm spectral and 50 ps temporal resolution
Source: Nat Commun. 2021 Nov 16;12:6616. doi: 10.1038/s41467-021-26837-0 (PMC8595732; doi:10.1038/s41467-021-26837-0)
Supplement: Supplementary file 3 — Description of Additional Supplementary Files [file 41467_2021_26837_MOESM3_ESM.pdf]

Description of Additional Supplementary Files

File name: Supplementary Movie 1
Description: Full spectrum fluorescence lifetime imaging of Convallaria Majalis. The movie shows a walk-through of the datacube with a colour representation of the intensity data, the corresponding lifetime image , weighted by the intensity, and the corresponding lifetime histogram for each wavelength.

File name: Supplementary Movie 2
Description: Full spectrum fluorescence lifetime imaging of a honeybee wing. The movie shows a walk-through of the datacube with the lifetime image for each wavelength, weighted by the intensity, and the corresponding lifetime histogram.
